# Tissue responses to everolimus-eluting stents implanted in severely calcified lesions following atherectomy

**DOI:** 10.1007/s12928-023-00965-4

**Published:** 2023-10-20

**Authors:** Tomohiro Yamaguchi, Takanori Yamazaki, Hisako Yoshida, Kotaro Matsumoto, Ryosuke Yahiro, Kazuhiro Nakao, Yusuke Kure, Tsukasa Okai, Takenobu Shimada, Kenichiro Otsuka, Yasuhiro Izumiya, Daiju Fukuda

**Affiliations:** 1 Department of Cardiovascular Medicine, Osaka Metropolitan University Graduate School of Medicine, 1-4-3 Asahimachi, Abeno-Ku, Osaka, 545-8585 Japan; 2 Department of Medical Statistics, Osaka Metropolitan University Graduate School of Medicine, Osaka, Japan; 3 Department of Cardiovascular Medicine, Kashiwara Municipal Hospital, Osaka, Japan; 4 Department of Cardiovascular Medicine, Ishikiri Seiki Hospital, Osaka, Japan

**Keywords:** Vascular healing, Neointimal coverage, Frequency-domain optical coherence tomography, Severe calcified coronary artery, Atherectomy

## Abstract

Histopathological examination has revealed that stents on severely calcified plaques were associated with delayed vascular healing. Although atherectomy devices can increase the number of malapposed struts, tissue responses to implanted drug eluting stents in atherectomy patients remain largely unknown. This retrospective observational study included 30 patients who underwent atherectomy and everolimus-eluting stent (EES) deployment for severely calcified coronary lesions (biodegradable polymer EES (BP-EES), *n = *15; durable polymer EES (DP-EES), *n*  = 15). Optical coherence tomography was carried out at baseline and follow-up, and struts with acute stent malapposition (ASM) were categorized as struts on modified calcium (mod-Ca), non-modified calcium (non-mod-Ca), or non-calcium (non-Ca). Adequate vascular healing, defined as ASM resolution with neointimal coverage, was compared between the BP-EES and DP-EES groups. Multivariate linear regression analysis using a generalized estimated equation revealed that BP-EES use was associated with significantly better adequate vascular healing compared with DP-EES (odds ratio [OR]: 3.691, 95% confidence interval [CI] 1.175–11.592, *P* = 0.025). adequate vascular healing was associated with the underlying plaque morphology (mod-Ca vs non-mod-Ca: OR 2.833, 95% CI 1.491–5.384, *P* = 0.001; non-Ca vs non-mod-Ca: OR 1.248, 95% CI 0.440–3.543, *P* = 0.677). This study demonstrates that drug-eluting stent selection and calcium modification are possible factors affecting vascular healing of malapposed struts in severely calcified lesions.

**Graphical Abstract:**

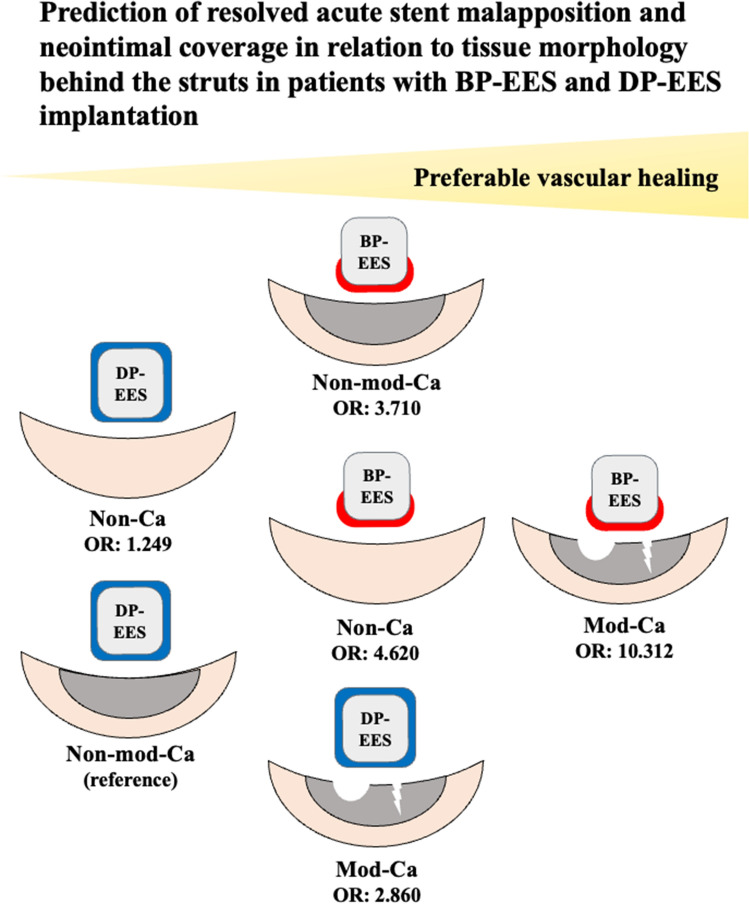

## Introduction

Severely calcified coronary stenosis is a challenging issue in percutaneous coronary intervention (PCI) because of its high rate of adverse cardiovascular events, even after drug-eluting stent (DES) implantation [1–4]. A recent histopathological study demonstrated that the stent struts on severely calcified plaques showed delayed vascular healing compared with non-severely calcified plaques, resulting in more uncovered struts in patients with severely calcified coronary arteries [5]. Uncovered struts have been associated with acute, subacute, and late stent thrombosis, and are thus an important issue in the management of patients with DES implantation [6]. In addition, uncovered struts were a major cause of very late stent thrombosis in patients without neoatherosclerosis [7].

Atherectomy devices, such as rotational and orbital atherectomy, are clinically useful for reducing the calcium volume and providing optimal stent expansion. However, aggressive strategies using atherectomy devices may result in fractured or scraped calcium modification, which may in turn lead to increased numbers of malapposed struts after DES implantation [8]. Such malapposed struts present a potential cause of delayed vascular healing, resulting in an increased number of uncovered struts [5, 8]. A previous study that analyzed vascular healing after rotational atherectomy using frequency-domain optical coherence tomography (FD-OCT) showed that some acute stent malpositions (ASM) could be resolved in the mid-term follow-up [8]. It has also been reported that tissue responses to ASM are heterogeneous, depending on the underlying tissue composition behind the struts and the distance between the stent struts and vessel wall ( S–V distance).

It has been reported that current-generation DES shows accelerated vascular healing, possibly due to developing technologies including biodegradable polymers, abluminal coating techniques, and thinner stent struts [9–11]. These biocompatible technologies can effectively promote vascular healing, even on severely calcified plaques. However, little is known about the efficacy of these technologies in patients with severely calcified coronary stenosis requiring atherectomy.

This study aimed to investigate the vascular healing of malapposed struts in patients with severely calcified coronary stenosis requiring atherectomy and treated with abluminal biodegradable polymer everolimus-eluting stents (BP-EES) or durable polymer everolimus-eluting stents (DP-EES).

## Methods

### Study population and protocol

The study protocol was carried out in accordance with the Declaration of Helsinki and was approved by Ethical Committee of Osaka Metropolitan University Graduate School of Medicine (Approval Number: 2020–271). This retrospective observational study enrolled 31 patients, treated at Osaka Metropolitan University Hospital from June 2015 to June 2021, who fulfilled the following eligibility criteria: (1) patients who underwent PCI using FD-OCT, (2) patients with severely calcified coronary stenosis (calcium arc > 180°), (3) patients who underwent PCI using atherectomy devices (rotational or orbital atherectomy), (4) patients who underwent BP-EES (Synergy, Boston Scientific, Marlborough, MA, USA) or DP-EES (Xience, Abbott Vascular, Santa Clara, CA, USA) implantation, and (5) patients who underwent follow-up coronary angiography and FD-OCT observation of the target vessels in 180 to 730 days after EES implantation, aiming to evaluate the early to mid-term vascular healing. Patients with acute thrombosis in the culprit lesion after PCI were excluded (*n = *1). A total of 30 patients with 30 lesions were finally included in the statistical analysis.

### PCI procedures and FD-OCT analysis

PCI procedures were performed using standard techniques and the atherectomy devices were used according to current guidelines [12–14]. After the 0.014-in. guide wire passed the target lesion, the lesion morphology was evaluated by FD-OCT (Lunawave OFDI® imaging system, Terumo, Tokyo, Japan; or ILUMIEN OPTIS® imaging system, Abbott Vascular). FD-OCT observations were also performed after DES implantation to evaluate the extent of stent malaposition and stent expansion. The stents and atherectomy devices were selected at the operators’ discretion. Rotational atherectomy (Rotablator® or RotaPro®, Boston Scientific, Natick, MA, USA) was performed using a solution of saline mixed with heparin, verapamil, nicorandil, and nitroglycerin. Orbital atherectomy was performed using a Diamondback 360® coronary orbital atherectomy system (Cardiovascular Systems, Inc., St. Paul, MN, USA). Vascular healing was assessed by follow-up coronary angiography using FD-OCT, at times determined by the attending physician.

**Fig. 1:**
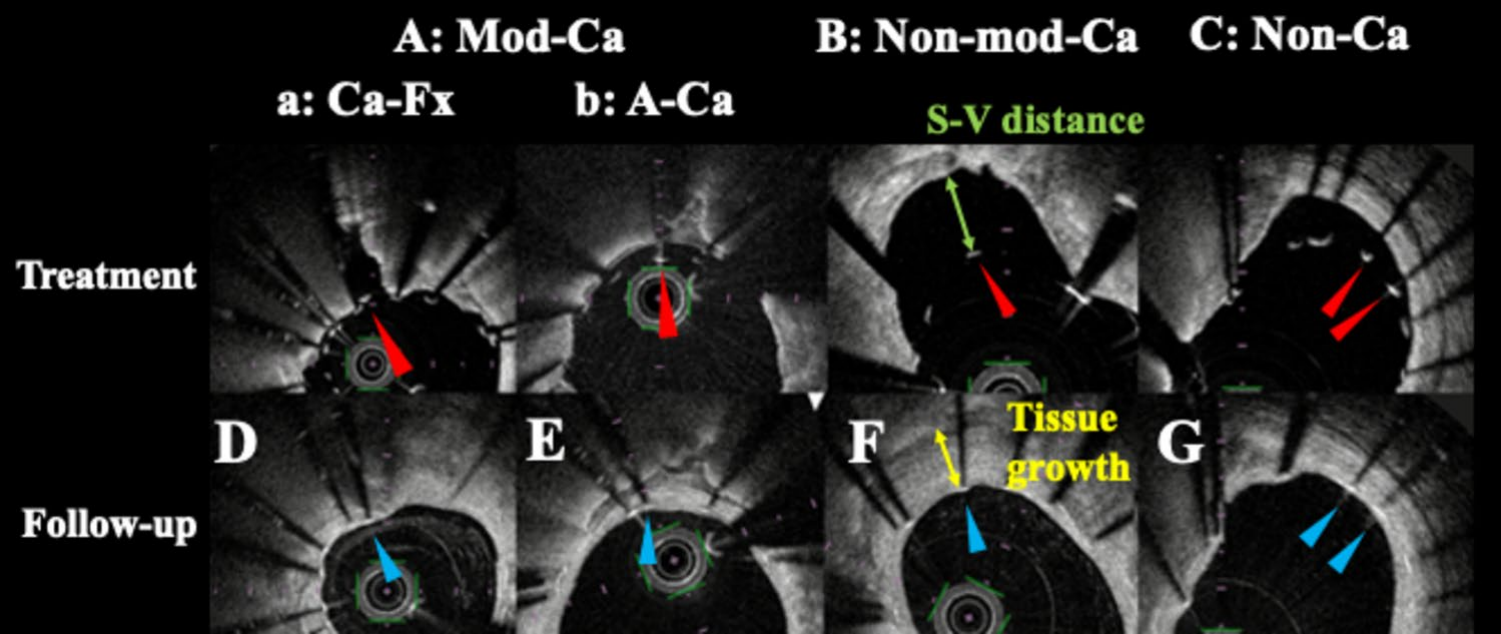
Representative paired cross-section of frequency-domain optical coherence tomography images at baseline and follow-up. **a**–**c** Classification of malapposed struts on background plaque morphology at baseline. **a** Malapposed strut on fractured calcium or calcium modified with atherectomy device (modified calcium; mod-Ca); **a** malapposed strut on fractured calcium (Ca-Fx; red arrow); **b:** malapposed strut on calcium modified with atherectomy device (A-Ca; red arrow). **b** Malapposed strut on calcium without fracture or modification with atherectomy (non-mod-Ca; red arrows). **c** Malapposed struts on non-calcium tissue (non-Ca; red arrows). **d**–**g** Matched struts with resolved acute stent malposition and neointimal coverage at follow-up (blue arrows)

**Fig. 2:**
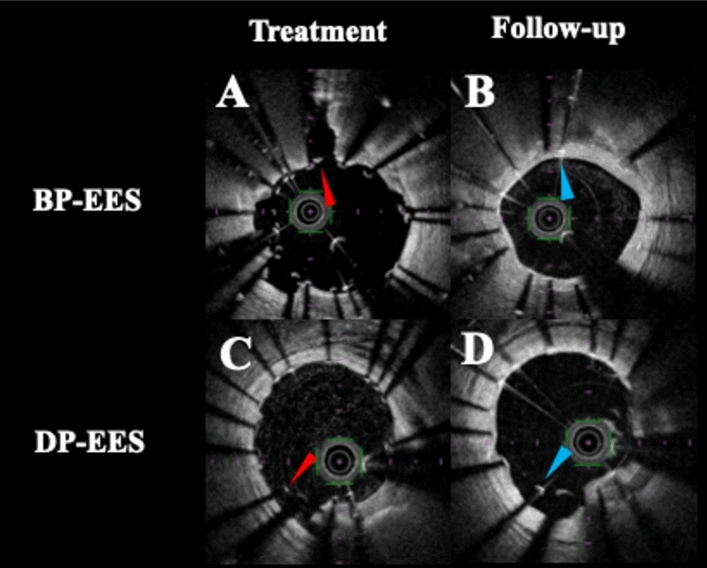
Representative paired cross-sectional images of BP-EES and DP-EES. **a** Cross-sectional image of implanted BP-EES at baseline. Red arrow indicated a malapposed strut of Mod-Ca. **b** Cross-sectional image of implanted BP-EES at follow-up. Adequate vascular healing was observed (blue arrow). **c** Cross-sectional image of implanted DP-EES at baseline. Red arrow indicated a malapposed strut of Non-Ca. **d** Cross-sectional image of implanted DP-EES at follow-up. Resolution of stent malapposition was not observed (blue arrow)

Baseline and follow-up FD-OCT images were co-registered based on the side branches, plaque morphologies, and lumen shapes, and struts were detected and measurements were assessed at fixed intervals of 1 mm from the distal to proximal stent edge. The S–V distance (μm) was measured as the length between the luminal edge of the strut surface and the vessel wall. ASM was defined as S–V distance  ≥ 200 μm [8]. Struts with ASM were categorized according to the plaque morphology behind the struts as follows: (1) struts on fractured calcium or calcium modified with atherectomy device (modified calcium; mod-Ca), (2) struts on calcium without fracture or modification with atherectomy (non-modified calcium; non-mod-Ca), and (3) struts on non-calcium tissue (non-calcium; non-Ca). Mod-Ca was additionally classified into struts on fractured calcium (Ca-Fx) and struts on calcium modified with atherectomy device (A-Ca) (Figs. 1, 2) [8]. ASM resolution, neointimal coverage, residual S–V distance, neointimal thickness (NIT; µm), and tissue growth (µm) were evaluated on follow-up FD-OCT images. ASM resolution was defined as a residual  S–V distance ≤ 0 mm at follow-up. Adequate vascular healing was defined as struts with ASM resolution and neointimal coverage. NIT was measured as the distance from the vessel wall to the luminal edge of the stent struts in FD-OCT images. Tissue growth was defined as the change in length from baseline vessel wall to the follow-up vessel wall via the struts, calculated from S–V distance, neointimal thickness, and residual S–V distance.

The plaque morphology behind the struts and adequate vascular healing were analyzed independently by two observers who were blinded to the patient information. In the event of disagreement between the two observers, the FD-OCT images were examined by a third observer and the final decisions were based on the majority results. NIT and tissue growth were calculated as the mean values of the two observers’ measurements.

### Statistical analysis

Continuous variables were summarized as median and interquartile range (quartiles 1–3) and categorical variables were summarized as number and percentage. Differences in continuous variables between the BP-EES and DP-EES groups were compared using Mann–Whitney *U* tests, and differences in categorical variables were compared using Fisher’s exact tests. Variables associated with vascular healing were identified by multiple linear regression analyses employing the following variables: stent type (BP-EES or DP-EES), S–V distance, cross-sectional lumen area (mm^2^), follow-up duration (days), and plaque morphology behind the struts, which were considered to be variables with possible clinical associations with vascular healing [8]. To account for clustering by duplication of patient information, we used a regression model with a generalized estimating equation to predict adequate vascular healing, in which the effect of continuous variables was estimated per 1-standard deviation increase. Mixed-effect models were employed to predict NIT and tissue growth. Struts in small lumens (lumen area < 3 mm^2^) at follow-up were eliminated as restenosed struts in the statistical analysis [8]. NIT was evaluated excluding struts without resolution of ASM.

Statistical analyses were performed using IBM SPSS software version 27 (IBM Co., Armonk, NY, USA) and R 4.1.2 (R Foundation for Statistical Computing, Vienna, Austria). The significance level was set at *P *< 0.05, and the alternative hypothesis was two-sided. Intraobserver and interobserver reproducibility were analyzed using linear weighted kappa statistics.

## Results

### Patient characteristics

The patients’ characteristics at the PCI procedure are listed in Table 1. Prescriptions and laboratory data for the study patients are shown in Table 2. Twenty-three patients (76.6%) received dual anti-platelet therapy and six patients (20.0%) used oral anticoagulants for atrial fibrillation. All of patients who used oral anticoagulants were prescribed single anti-platelet therapy. A patient received initial single anti-platelet therapy due to history of severe intestinal hemorrhage. There was not significant difference in dual anti-platelet therapy duration between BP-EES and DP-EES group. There were no significant differences in pre- and post-procedural laboratory data between the BP-EES and DP-EES groups, except for serum triglyceride levels.

**Table 1:** Patients’ clinical characteristics

Parameters	Total (*n = *30)	BP-EES (* n* = 15)	DP-EES (*n = *15)	*P*-value
Age, years	77 (74–80)	78 (75–81)	77 (70–79)	0.197
Male	22 (73.3)	11 (73.3)	13 (73.3)	1.000
BSA, m^2^	1.63 (1.50–1.77)	1.62 (1.48–1.76)	1.65 (1.56–1.75)	0.852
Clinical presentation				
SAP	13 (43.3)	8 (53.3)	5 (33.3)	0.065
SMI	13 (43.3)	4 (26.7)	9 (60.0)	
UAP	3 (10.0)	3 (20.0)	0 (0)	
NSTEMI	1 (3.3)	0 (0)	1 (6.7)	
Target lesions				
LAD	23 (76.7)	10 (66.7)	13 (86.7)	0.477
LCx	2 (6.7)	2 (13.3)	0 (0)	
RCA	5 (16.7)	3 (20.0)	2 (13.3)	
Atherosclerotic risks				
Hypertension	25 (83.3)	13 (86.7)	12 (80.0)	1.000
Dyslipidemia	24 (80.0)	10 (66.7)	14 (93.3)	0.169
Diabetes mellitus	14 (46.7)	4 (26.7)	10 (66.7)	0.066
Current smoking	13 (43.3)	5 (33.3)	8 (53.3)	0.462
Past history				
Previous PCI	10 (33.3)	5 (33.3)	5 (33.3)	1.000
Previous CABG	0 (0)	0 (0)	0 (0)	NA
Previous cerebral infarction	4 (13.3)	1 (6.7)	3 (20.0)	0.598
Atrial fibrillation	7 (23.3)	4 (26.7)	3 (20.0)	1.000
Hemodialysis	1 (3.3)	0 (0)	1 (6.7)	1.000

Data presented as *n* (%) or interquartile range (25%–75%)

*BSA*  body surface area, *SAP* stable angina pectoris, *SMI* silent myocardial ischemia, *UAP* unstable angina pectoris, *NSTEMI* non-ST-elevated myocardial infarction, *PCI*  percutaneous coronary intervention, *CABG* coronary artery bypass grafting

**Table 2:** Prescriptions and laboratory data before and after procedure

Parameters	Total (*n = *30)	BP-EES (*n = *15)	DP-EES (*n = *15)	*P*-value
Prescriptions				
DAPT	23 (76.6)	11 (73.3)	12 (80.0)	1.000
Duration of DAPT, days	354 (319–407)	364 (340–407)	343 (259–410)	0.278
Cilostazol	1 (3.3)	0 (0)	1 (6.7)	1.000
Aspirin	23 (76.7)	11 (73.3)	12 (80.0)	1.000
Clopidogrel	19 (63.3)	9 (60.0)	10 (66.7)	1.000
Prasugrel	10 (33.3)	6 (40.0)	4 (26.7)	0.700
Oral Anticoagulants	6 (20.0)	3 (20.0)	3 (20.0)	1.000
ACE-i/ARB	18 (60.0)	9 (60.0)	9 (60.0)	1.000
β-blocker	13 (43.3)	7 (46.7)	6 (40.0)	1.000
Statin	28 (93.3)	14 (93.3)	14 (93.3)	1.000
Laboratory data				
Pre-procedure				
eGFR, mL/min per 1.73 m^2^	63.2 (51.2–73.3)	62.4 (52.8–75.9)	64.0 (50.3–72.9)	0.624
Hs-CRP, mg/dL	0.11 (0.03–0.29)	0.17 (0.07–0.29)	0.06 (0.03–0.21)	0.253
TG, mg/dL	98 (73–119)	71 (62–150)	108 (97–131)	0.006
T.Cho, mg/dL	147 (126–164)	140 (125–161)	150 (130–169)	0.372
HDL-C, mg/dL	47 (34–58)	49 (36–56)	44 (34–71)	0.983
LDL-C, mg/dL	77 (67–99)	77 (69–95)	78 (68–99)	0.930
HbA1c, %	6.3 (5.9–6.7)	6.0 (5.8–6.4)	6.4 (6.1–6.9)	0.084
BS, mg/dL	100 (92–119)	94 (86–103)	113 (98–131)	0.044
CK, U/L	95 (64–165)	92 (72–154)	97 (58–180)	0.934
CK-MB, U/L	8 (7–11)	8 (7–13)	9 (8–11)	0.834
Hs-TnT, ng/mL	0.015 (0.008–0.024)	0.016 (0.009–0.021)	0.014 (0.009–0.050)	0.868
BNP, pg/mL	66.7 (17.0–114.8)	82.1 (38.8–110.0)	26.6 (15.2–103.9)	0.123
Post-procedure				
eGFR, mL/min per 1.73 m^2^	60.6 (52.0–74.9)	57.4 (47.2–75.6)	61.8 (55.7–72.6)	0.595
CK, U/L	118 (87–157)	119 (91–143)	103 (85–172)	1.000
CK-MB, U/L	10 (7–15)	12 (8–15)	10 (7–14)	0.662
Hs-TnT, ng/mL	0.103 (0.070–0.187)	0.137 (0.085–0.190)	0.098 (0.057–0.142)	0.266

Data presented as n (%) or interquartile range (25%–75%)

*DAPT*  dual anti-platelet therapy, *ACE-I* angiotensin converting enzyme inhibitor, *ARB*   angiotensin II receptor blocker, *eGFR*   estimated glomerular filtration rate, *Hs-CRP* high sensitivity C-reactive protein, *TG* triglyceride, *T.Cho*  total cholesterol, *HDL-C* high-density lipoprotein cholesterol, *LDL-C* low-density lipoprotein cholesterol, *HbA1c* hemoglobin A1c, *BS* blood sugar, *CK* creatine kinase, *CK-MB* creatine kinase MB, *Hs-TnT* high-sensitivity troponin T, *BNP* brain natriuretic peptide

### Lesion characteristics and procedures

Lesion characteristics assessed by FD-OCT are shown in Table 3. The median calcium arc was 293.8° (253.8–326.0°) and the median calcium thickness was 1.35 mm (1.15–1.60 mm). Five patients required lesion modification using atherectomy devices before initial FD-OCT observation because the imaging catheter could not pass the severe stenosis. Rotational atherectomy was used for lesion modification in 26 patients (86.7%) and orbital atherectomy was used in the other patients. The total length of the deployed stents was 32 mm (23–38 mm) and the median final TIMI flow grade was 3 (3–3). Two patients had reduced TIMI flow (grade 2) at final angiography.

**Table 3:** Lesion characteristics, procedural data, and FD-OCT analysis at PCI

Parameters	Total (*n = *30)	BP-EES (*n = *15)	DP-EES (*n = *15)	*P*-value
Lesion length, mm	27.1 (19.0–36.6)	26.2 (19.6–35.2)	29.6 (21.4–36.7)	0.494
Minimal lumen area, mm^2^	1.60 (1.36–2.08)	1.60 (1.52–2.25)	1.70 (1.28–2.05)	0.835
Reference vessel diameter, mm	2.57 (2.29–2.86)	2.56 (2.29–2.81)	2.57 (2.30–2.96)	0.917
Calcium arc, °	293.8 (253.8–326.0)	301.0 (291.4–349.8)	268.5 (242.5–301.8)	0.088
Maximum calcium thickness, mm	1.35 (1.15–1.60)	1.41 (1.20–1.67)	1.34 (1.13–1.48)	0.345
Atherectomy device				
Rotational	26 (86.7)	14 (93.3)	12 (80.0)	0.598
Orbital	4 (13.3)	1 (6.7)	3 (20.0)	
Number of stents	1 (1–1)	1 (1–2)	1 (1–1)	0.386
Distal stent diameter, mm	3.00 (2.75–3.00)	3.00 (2.75–3.25)	3.00 (2.75–3.00)	0.441
Total stent length, mm	32 (23–38)	24 (22–46)	33 (29–38)	0.479
Final TIMI flow grade	3 (3–3)	3 (3–3)	3 (3–3)	0.164
Minimal stent area, mm^2^	5.64 (4.52–6.85)	6.07 (5.51–7.11)	4.89 (4.10–5.88)	0.045
Analyzed cross-section	956	488	468	–
Analyzed cross-section per patient	31 (24–38)	28 (24–38)	33 (25–37)	0.901
Aanalyzable cross-section	928	479	449	–
Aanalyzable cross-section per patient	29 (24–37)	28 (23–37)	29 (25–36)	0.917
Analyzable struts	6234	3053	3181	–
Analyzable struts per patient	200 (140–268)	189 (136–263)	226 (151–270)	0.575
Analyzable struts/cross-section	6.6 (5.5–7.6)	5.9 (5.5–6.9)	6.8 (6.4–7.9)	0.161
Mean S–V distance, μm	72 (50–101)	68 (47–103)	78 (57–100)	–
Malapposed struts	401	243	158	–
Malapposed struts per patient	10 (3–14)	10 (7–19)	6 (3–14)	0.271
S–V distance of malapposed struts, μm	289 (237–392)	295 (242–394)	282 (233–360)	–
Plaque morphology behind the malapposed struts				
Mod-Ca	92 (22.9)	49 (20.2)	43 (27.2)	0.053
Ca-Fx	27 (6.9)	11 (4.5)	17 (10.8)	
A-Ca	63 (16.1)	38 (15.6)	26 (16.5)	
Non-mod-Ca	107 (26.7)	60 (24.7)	47 (29.7)	
Non-Ca	202 (50.4)	134 (55.1)	68 (43.0)	

Data presented as n (%), interquartile range (25%–75%)

*S–V distance*  stent–umen distance, *Mod-Ca* modified calcium, *Ca-Fx* fractured calcium, *A-Ca* atherectomy calcium, *Non-mod-Ca* non-modified calcium, *non-Ca*   non-calcium

### FD-OCT analysis of deployed stents at baseline

The baseline FD-OCT parameters are shown in Table 3. A total of 6234 paired struts in 956 cross-sections were detected in baseline FD-OCT images. The median S–V distance of the detected struts was 72 μm (50–101 μm), 401 struts (6.4%) were malapposed, and the median S–V distance of these malapposed struts was 289 μm (237–392 μm).

Regarding the classification of plaque morphology behind the struts, 92 struts (22.9%) were categorized as mod-Ca, 107 (26.7%) as non-mod-Ca, and 202 (50.4%) as non-Ca. In addition, 108 mod-Ca struts were categorized as Ca-Fx (27 struts; 6.9%) or A-Ca (63 struts; 16.1%). Intraobserver reproducibilities for interpreting plaque morphology behind the struts were 95.9% and 93.4%, respectively, and the interobserver reproducibility was 86.8%.

### Follow-up data and follow-up FD-OCT analysis.

Follow-up data are shown in Table 4. The median follow-up duration was 351 days (284–407 days). One patient underwent revascularization at follow-up due to restenosis of the stent edge. Of the 401 malapposed struts, 31 (7.7%) were excluded from the statistical analysis because of unclear images induced by inadequate blood flush or wire shadow at follow-up FD-OCT. Nine struts (2.2%) were eliminated because of a restenosed cross-section (lumen area < 3.0 mm^2^). Finally, 361 analyzable paired struts were detected in initial and follow-up FD-OCT images, including 308 struts (85.3%) with resolved ASM and median residual S–V distance 311 μm (149–420 μm). In addition, 311 of the 361 struts (86.2%) were covered with neointima and 301 (83.4%) had adequate vascular healing. The median neointimal thickness was 64 μm (36–132 µm). The intraobserver reproducibilities for interpreting resolution of ASM were 90.9% and 93.6%, respectively, and the interobserver reproducibility was 82.7%. The intraobserver reproducibilities for interpreting neointimal coverage were 88.7% and 93.6%, respectively, and the interobserver reproducibility was 77.7%.

**Table 4:** Follow-up FD-OCT analysis of acute malapposed struts

Parameters	Total (*n = *30)	BP-EES (*n = *15)	DP-EES (*n = *15)	*P*-value
Follow-up duration, days	351 (284–407)	357 (329–396)	349 (267–417)	0.917
Revascularization	1 (3.3)	0 (0)	1 (6.7)	1.000
Analyzable struts	361 (90.0)	222 (91.4)	139 (88.0)	–
Struts without analyzable FD-OCT images	31 (7.7)	21 (8.6)	10 (6.3)	–
Struts in restenosed cross-section	9 (2.2)	0 (0)	9 (5.7)	–
Resolved ASM / analyzable struts	308 (85.3)	204 (91.9)	104 (74.8)	< 0.001
Residual S–V distance of struts without resolution of ASM, µm	311 (149–420)	295 (205–450)	330 (185–397)	0.425
Neointimal coverage/ analyzable struts	311 (86.2)	205 (92.3)	106 (76.3)	< 0.001
Neointimal thickness, µm	64 (36–132)	58 (32–130)	75 (44–150)	0.028
Adequate vascular healing/analyzable struts	301 (83.4)	199 (89.6)	102 (73.4)	< 0.001
Tissue growth, µm	364 (277–490)	393 (291–517)	330 (242–468)	0.003

Data presented as n (%), interquartile range (25%–75%)

*ASM*  acute stent malposition, *S–V distance* stent–vessel lumen distance

### Effects of plaque morphologies and DES type to the tissue response.

**Fig. 3:**
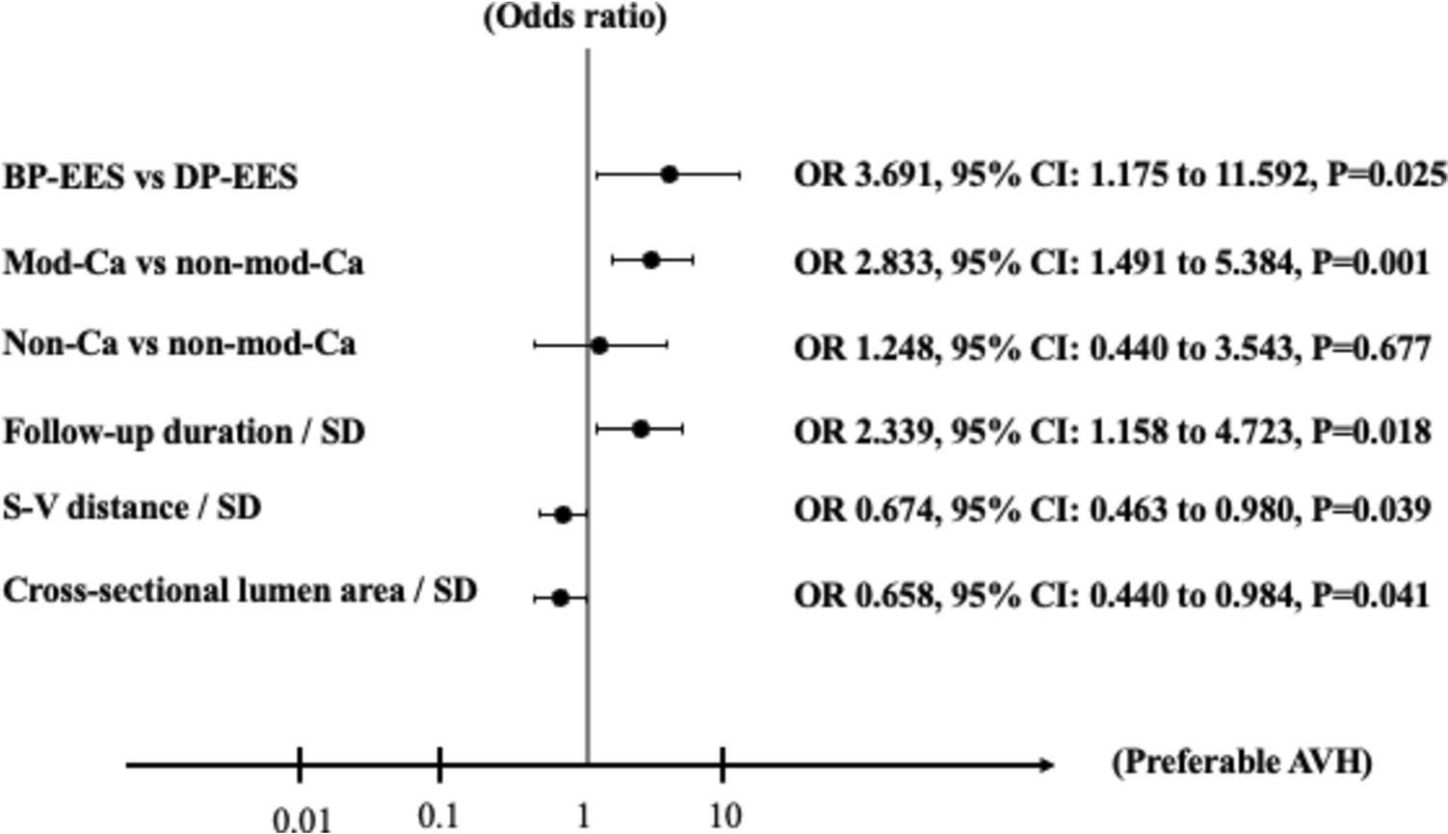
The estimated effect of calcium modification and stent selection to predict the adequate vascular healing using multivariate linear regression analysis via generalized estimated equation. Continuous variables were employed to estimate adequate vascular healing as 1-standard deviation (SD) increase in this model. The SDs for each parameter in malapposed struts were 114 days for follow-up duration, 141 μm for S–V distance, and 2.91 mm^2^ for cross-sectional lumen area. *BP-EES*   biodegradable polymer everolimus-eluting stent, *DP-EES*  durable polymer everolimus-eluting stent, *mod-Ca* modified calcium, *non-mod-Ca*   non-modified calcium, *non-Ca* non-calcium, *S–V distance* stent–vessel lumen distance

**Fig. 4:**
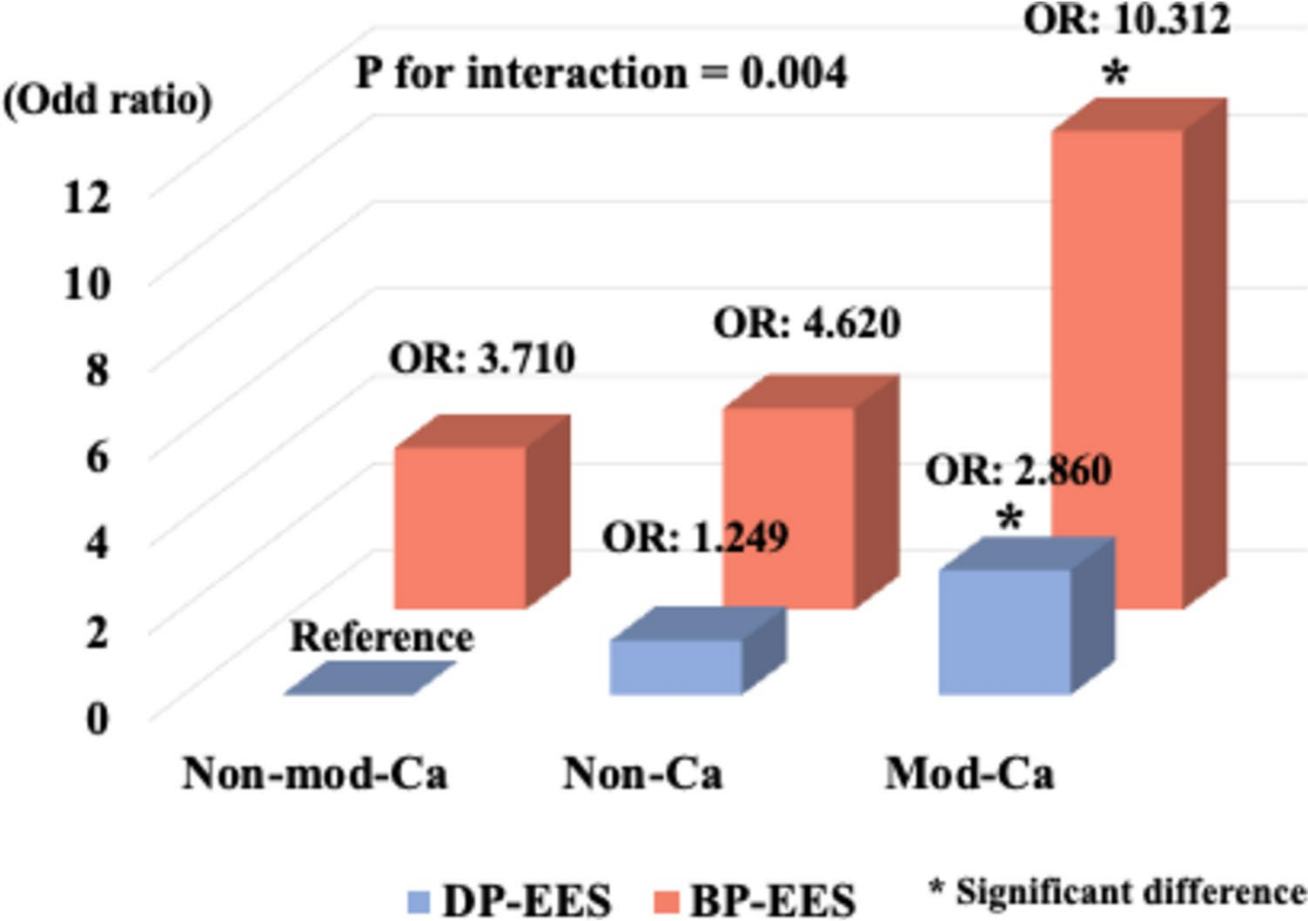
Effect modification model predicting adequate vascular healing of plaque morphologies and stent type using regression analysis via generalized estimating equation, adjusting for follow-up duration, S–V distance, and cross-sectional lumen area. Adequate vascular healing was significantly associated with stent type and plaque morphology behind the struts, comparing DP-EES with non-mod-Ca as reference (BP-EES on mod-Ca: OR 10.312, 95% CI 2.211–48.090, *P* = 0.003; BP-EES on non-Ca: OR 4.620, 95% CI 0.885–24.114, *P*  = 0.069; BP-EES on non-mod-Ca: OR 3.710, 95% CI 0.629–21.877, *P*  = 0.148; DP-EES on mod-Ca: OR 2.860, 95% CI 1.226–6.672, *P*  = 0.015; DP-EES on non-Ca: OR 1.249, 95% CI 0.357–4.374, *P*  = 0.728; *P* for interaction  = 0.004)

Multivariate linear regression analysis using a generalized estimated equation revealed that adequate vascular healing was significantly better in patients who received BP-EES compared with those who received DP-EES (odds ratio [OR] 3.691, 95% confidence interval [CI] 1.175–11.592, P = 0.025) (Fig. 3). Follow-up duration, S–V distance, and cross-sectional lumen area were also significant factors associated with adequate vascular healing. Additionally, adequate vascular healing was associated with the plaque morphology behind the struts (mod-Ca vs non-mod-Ca: OR 2.833, 95% CI 1.491–5.384, *P* = 0.001; non-Ca vs non-mod-Ca: OR 1.248, 95% CI 0.440–3.543, *P* = 0.677). In a regression model adjusting for follow-up duration, S–V distance, and cross-sectional lumen area, adequate vascular healing was significantly associated with DES type and plaque morphology behind the struts, while adequate vascular healing was best for mod-Ca with BP-EES (*P* for interactio*n = *0.004) (Fig. 4).

Additional analysis of the detailed classification of plaque morphology behind the struts identified stent type, follow-up duration, S–V distance, cross-sectional lumen area as being significantly associated with adequate vascular healing (BP-EES vs DP-EES: OR 3.832, 95% CI 1.207–12.168, *P* = 0.023; follow-up duration: OR 2.311, 95% CI 1.174–4.548, *P*  = 0.015; S–V distance: OR 0.645, 95% CI 0.433–0.961, *P*  = 0.031; cross-sectional lumen area: OR 0.665, 95% CI 0.444–0.996, *P* = 0.048). Plaque morphology behind the struts was significantly associated with adequate vascular healing (Ca-Fx vs non-mod-Ca: OR 4.654, 95% CI 1.600–13.539, *P* = 0.005; A-Ca vs non-mod-Ca: OR 2.216, 95% CI 1.099–4.469, *P* = 0.026; non-Ca vs non-mod-Ca: OR 1.229, 95% CI 0.427–3.542, *P* = 0.702).

In struts with neointimal coverage (311 struts), the regression model demonstrated no significant association with NIT among stent types, plaque morphologies behind the struts, S–V distance, and cross-sectional lumen area, whereas follow-up duration was significantly associated with NIT (Table 5). However, follow-up duration and S–V distance were significantly associated with tissue growth in a mixed-effect model (Table 5). Stent type and cross-sectional lumen area could not significantly predict tissue growth. Plaque morphology behind the strut was significantly associated with tissue growth (mod-Ca vs non-mod-Ca; *B* = 79.7, 95% CI 13.4–146.0, *P* = 0.019; non-Ca vs non-mod-Ca: *B* = 21.9, 95% CI  − 22.5 to 66.4, *P* = 0.333).

**Table 5:** Mixed-effect model to predict NIT and tissue growth

	B	95% CI	*P*-value
NIT			
BP-EES vs DP-EES	−28.7	−82.1 to 24.7	0.291
Follow-up duration, days	24.3	4.1 to 44.5	0.018
Plaque morphologies behind the malapposed struts			
Mod-Ca vs non-mod-Ca	32.9	−40.9 to 106.7	0.381
Non-Ca vs non-mod-Ca	7.5	−33.6 to 48.7	0.719
S–V distance, mm	−12.4	−32.5 to 7.8	0.227
Cross-sectional lumen area, mm^2^	0.04	−17.9 to 18.0	0.996
Tissue growth			
BP-EES vs DP-EES	38.2	−42.4 to 118.8	0.352
Follow-up duration, days	66.5	26.6 to 106.3	0.001
Plaque morphologies behind the malapposed struts			
Mod-Ca vs non-mod-Ca	79.7	13.4 to 146.0	0.019
Non-Ca vs non-mod-Ca	21.9	−22.5 to 66.4	0.333
S–V distance, mm	103.3	71.1 to 135.5	< 0.001
Cross-sectional lumen area, mm^2^	−11.1	−39.0 to 16.8	0.435

*BP-EES*   biodegradable polymer everolimus-eluting stent, *DP-EES*   durable polymer everolimus-eluting stent, *mod-Ca*  modified calcium, *non-mod-Ca* non-modified calcium, *non-Ca* non-calcium, *S–V distance* stent–vessel lumen distance

## Discussion

The present study demonstrated the difference in vascular healing of malapposed struts among patients with severely calcified coronary arteries requiring atherectomy devices, and treated with different EES types. The major findings of the present study were that struts on mod-Ca had better vascular healing, and BP-EES implantation was associated with better adequate vascular healing compared with DP-EES. To the best of our knowledge, there have been a few previous reports of vascular healing analyzed by FD-OCT after DES implantation in cases of severely calcified coronary stenosis [8].

DES with different features, such as coated drugs, polymer excipients, and coating techniques, have been clinically available for the treatment of coronary stenosis [9–11]. Although the Synergy BP-EES and Xience DP-EES include the same amount of everolimus (1 μg/mm^2^), these DES use different coated polymers and coating techniques: the Xience is coated entirely with a durable antithrombogenic polymer, whereas the Synergy has abluminal coating with biodegradable polymers, most likely resulting in accelerated vascular healing [9, 10]. Some reports demonstrated similar clinical outcomes between BP-EES and DP-EES [15, 16]. However, the distinct tissue responses to BP-EES and DP-EES implantation in severely calcified lesions remain largely unknown.

Vascular healing after DES implantation begins with platelet aggregation and fibrin deposition with inflammatory cell infiltration, which attracts endothelial and smooth muscle cells [5]. It is therefore possible that an appropriate affinity to the thrombus may be important in the early stage of vascular healing after DES implantation, probably resulting in the better adequate vascular healing of BP-EES compared with DP-EES with antithrombotic polymer. Torii et al. reported that vascular healing was delayed in struts on calcified plaques, malapposed struts, and struts without medial tears [5]. In contrast, lumen loss was greater in struts with medial tears, which possibly provoked the vascular healing response resulting in excessive neointimal growth. Atherectomy is a reasonable strategy for reducing medial tears by facilitating balloon dilation of severely calcified stenosis without excessively high inflation pressure [5]. However, atherectomy itself may provoke excessive platelet activation on the surface of the calcium [17]. In the present study, mod-Ca tended to show better adequate vascular healing and tissue growth than non-mod-Ca, while NIT was similar in both EES groups. High platelet activation, possibly induced by atherectomy or calcium directly exposed to the vessel lumen itself, may have caused the better vascular healing in struts on mod-Ca. A previous pathological study in a rabbit model demonstrated that BP-EES was associated with better neointimal coverage, less inflammation, and less foamy macrophage infiltration within the neointima than a durable polymer zotarolimus-eluting stent [18]. BP-EES had the potential of preventing excessive neointimal growth, even on mod-Ca with provoked platelet aggregation followed by excessive inflammation.

The present study had several limitations. First, the small study population (*n = *30) might have resulted in weak statistical findings. Second, this was a single-center, retrospective observational study and we could therefore not exclude selection bias because of the highly selected study population. Third, it is possible that FD-OCT-based adequate vascular healing differed from pathologically preferable vascular healing. Finally, the current model did not consider atherosclerotic factors other than the data from the imaging catheter. Nevertheless, the present results further our understanding of the factors associated with vascular healing after DES implantation in severely calcified coronary arteries modified with atherectomy devices. However, further prospective trials are warranted to confirm our findings.

## Conclusions

There was a possibility that DES selection and calcium modification were factors affecting vascular healing after DES implantation in patients with severely calcified coronary arteries.
