# Correction: Tissue responses to everolimus-eluting stents implanted in severely calcified lesions following atherectomy

**DOI:** 10.1007/s12928-023-00973-4

**Published:** 2023-12-08

**Authors:** Tomohiro Yamaguchi, Takanori Yamazaki, Hisako Yoshida, Kotaro Matsumoto, Ryosuke Yahiro, Kazuhiro Nakao, Yusuke Kure, Tsukasa Okai, Takenobu Shimada, Kenichiro Otsuka, Yasuhiro Izumiya, Daiju Fukuda

**Affiliations:** 1 Department of Cardiovascular Medicine, Osaka Metropolitan University Graduate School of Medicine, 1-4-3 Asahimachi, Abeno-Ku, Osaka, 545-8585 Japan; 2 Department of Medical Statistics, Osaka Metropolitan University Graduate School of Medicine, Osaka, Japan; 3 Department of Cardiovascular Medicine, Kashiwara Municipal Hospital, Osaka, Japan; 4 Department of Cardiovascular Medicine, Ishikiri Seiki Hospital, Osaka, Japan

**Correction: Cardiovascular Intervention and Therapeutics** 10.1007/s12928-023-00965-4

In this article the graphic abstract was published incorrectly. The correct graphic abstract is given in this correction.

**Graphical Abstract**


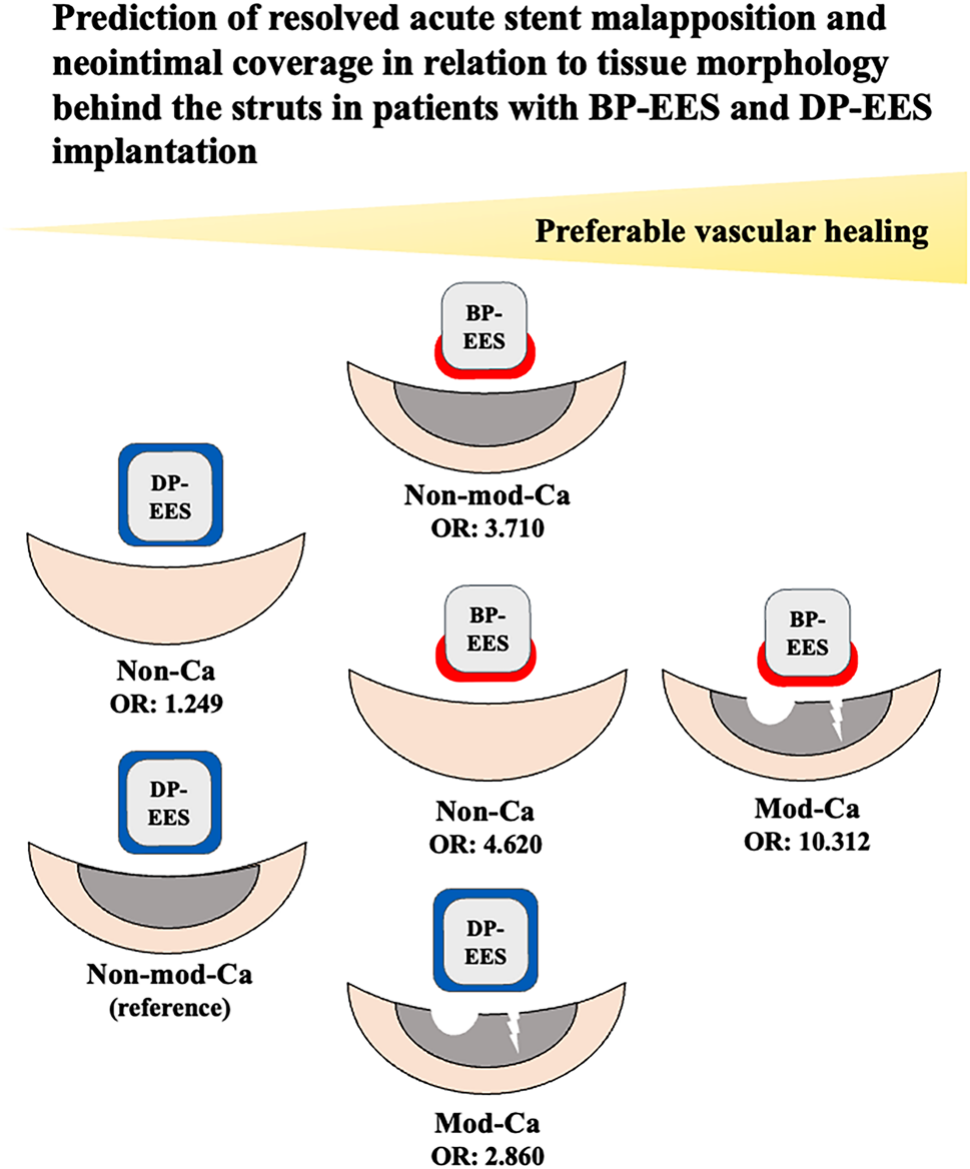


The original article has been corrected.

